# Enrollment, Childbearing Motivations, and Intentions of Couples in the Creighton Model Effectiveness, Intentions, and Behaviors Assessment (CEIBA) Study

**DOI:** 10.3389/fmed.2017.00147

**Published:** 2017-09-08

**Authors:** Joseph B. Stanford, Christina A. Porucznik

**Affiliations:** ^1^Department of Family and Preventive Medicine, University of Utah, Salt Lake City, UT, United States

**Keywords:** natural family planning, family planning, behavioral methods, cervical mucus method, knowledge, attitudes, practice, pregnancy intentions

## Abstract

**Context:**

The Creighton Model Fertility*Care*^TM^ System (CrM) is a standardized approach for educating women about the biomarkers of their fertility. Couples can use this information for timing intercourse during “fertile” or “infertile” days in order to try to conceive or to avoid pregnancy.

**Objectives:**

The study of Creighton Model Effectiveness, Intentions, and Behaviors Assessment (CEIBA) was conducted to assess fertility motivations, intentions, fertility-related sexual behaviors, and their impact on effectiveness to avoid and to conceive among new users of the CrM. This paper reports enrollment baseline characteristics.

**Settings and design:**

We conducted this prospective cohort study at 17 CrM Fertility*Care*^TM^ Centers; 16 in the USA and one in Toronto, Canada.

**Materials and methods:**

Couples who were new or returning users of the CrM were eligible. Couples who were initially trying to conceive or had a history of subfertility were excluded. Couples were enrolled and followed prospectively by their CrM instructors and also by CEIBA study staff. They completed baseline questionnaires.

**Results:**

1,132 new couples were assessed; 1,090 (96%) couples were screened; 429 (39%) couples were eligible; 305 women (71%) and 290 (95%) male partners were enrolled. The majority of women was engaged (39%) or married (51%), college graduates (77%), Caucasian non-Hispanic (80%), and Roman Catholic (80%). The most common reasons for learning CrM (women) were to use a natural method for family planning (91%), for moral/ethical/religious reasons (70%), the lack of side effects (71%), or insight into the menstrual cycle and fertility (62%). Women and men intended to have a mean of three and two additional children, respectively. Of women, 21% intended to have a child within a year and 60% between 1 and 3 years. The mean positive childbearing motivation score was 3.3 for both women and men (range 1–4, with 4 being most positive).

**Conclusion:**

Couples beginning use of the CrM to avoid pregnancy have high levels of motivation, desire, and intention for future childbearing. The CEIBA study has prospective measures of desires, intentions, and sexual/fertility behaviors for up to 1 year. We will assess the impact of desires, intentions, and behaviors on the pregnancy rates among these couples.

## Introduction

Methods of family planning based upon identifying the days of potential fertility in women, known as natural family planning (NFP) or fertility awareness-based methods (FABMs), are an important option for family planning (1–4). These methods use various biomarkers to identify days in the menstrual cycle when intercourse is likely to result in pregnancy. Among their advantages are low cost, lack of side effects, and the education they can provide users about their own reproductive physiology and health (5–7). By definition, NFP refers to avoiding pregnancy through abstinence from genital contact (8, 9), while FABM allows for the use of barriers or withdrawal on fertile days—as well as abstinence—when pregnancy avoidance is intended (10, 11).

In addition to their use in avoiding pregnancy, FABM or NFP methods are unique among family planning methods in their additional ability to help couples try to time pregnancy (12–14). Indeed, in population-based studies, potential interest in these methods has been greater for the purpose of conceiving than of avoiding conception (15, 16). Many, perhaps most, long-term users of FABM or NFP methods will at different times use the method both to avoid pregnancy and to conceive, transitioning between these intentions (17–19).

The Creighton Model Fertility*Care*^TM^ System (CrM), a NFP method based on initial research at St. Louis University, was further developed and implemented by Dr. Hilgers and colleagues at the Creighton University Medical Center and the Pope Paul VI Institute for the Study of Human Reproduction, Omaha, Nebraska (20, 21). It is based on standardized observation and recording of vaginal discharge (especially from cervical mucus) and vaginal bleeding, interpretation of these biomarkers for fertility and health status, and teaching using a case management approach (22). The CrM instructors are called Creighton Model Fertility*Care*^TM^ Practitioners (CrM FCPs). CrM instruction is provided through Creighton Fertility*Care*^TM^ Centers; in the United States and Canada, these are registered through Fertility*Care*^TM^ Centers of America, while in other parts of the world they are registered through Fertility*Care*^TM^ Centers International (www.fertilitycare.org). Additionally, a system of medical protocols called natural procreative technology (NaProTechnology) has been developed for supporting women with gynecologic problems and couples with infertility and integrated with the CrM (23). Integral to the teaching of the CrM is emphasizing to the couples that they have radical autonomy to choose to have intercourse or not on any given day with full knowledge of the possibility of pregnancy on that day (“selective intercourse”) and that there is no requirement for what they “should” do with regard to possible pregnancy outcomes (22).

Several previous studies have been published regarding the CrM’s effectiveness in both promoting or preventing pregnancy (24–30). These studies were based on assessments of the couples’ behavior and knowledge; they made very limited assessment of the relationships between intentions, behaviors, and pregnancy rates. The study of Creighton Model Effectiveness, Intentions, and Behaviors Assessment (CEIBA) was conducted to assess the spectrum of fertility motivations, intentions, and fertility-related sexual behaviors and quantify their impact on effectiveness in avoiding or achieving pregnancy among new users of the CrM with initial pregnancy avoidance intentions. In this paper, we report on the recruitment for CEIBA, baseline demographic characteristics, and the childbearing motivations and intentions of women and men (heterosexual couples) who started use of the CrM with the initial intention of avoiding pregnancy. We also describe the protocol for assessing follow-up intentions, behaviors, and pregnancy.

## Materials and Methods

### Design, Setting, Timeline, and Approval

The CEIBA study was a prospective cohort study implemented in 17 different CrM Fertility*Care*^TM^ Centers throughout the United States and Canada. Of the participating CrM centers, eight were directly connected to a Catholic institution, while the other nine were independent organizations, most with strong networking connections to local Catholic communities. Participating CrM Fertility*Care*^TM^ Centers and CrM FCPs are listed in the acknowledgments. All new or returning couples seeking instruction in the CrM were screened for study eligibility with a screening questionnaire. The study began enrollment in September 2009 and closed enrollment in December 2011. Some of the participating CrM Fertility*Care*^TM^ centers joined the study later, after September 2009; all finished enrollment by December 2011. The study was reviewed and approved by the University of Utah Institutional Review Board (IRB) number 00034487. All local participating sites either had local IRB approval or an authorization agreement relying upon the University of Utah IRB.

### Recruitment

Couples were recruited from among those normally presenting to the participating centers to learn the CrM. Participating CrM Fertility*Care*^TM^ Centers used posters and brochures to inform their clients and potential clients about the study. In addition, we recruited online through blog posts, websites, and a Facebook page. Inquiries from the online recruiting were referred to a participating CrM Fertility*Care*^TM^ Center for CrM instruction and study screening.

### Eligibility and Consent

We attempted to screen all couples presenting to the participating FCPs for eligibility. Initially, couples were eligible for this study if they were learning CrM for the first time with the stated intention of avoiding pregnancy. Screening for eligibility was performed by the CrM FCPs, using a screening questionnaire. Specific exclusion criteria are listed in Table 1. These were designed to exclude couples who had possible reduced fecundity, had previously used the CrM, were not planning to be sexually active within 6 months, could not communicate adequately to complete study procedures, or were under 18 years of age.

**Table 1:** Reasons and frequencies for exclusion from the CEIBA study (*n* = 704).

Eligibility unknown	*N* (%)
Refused screening	37 (5.3)
Screening not completed	6 (0.9)

**Ineligible^a^**	

Could not speak English	1 (0.1)
Woman < 18 years old	10 (1.4)
Woman > 40 years old	81 (11.5)
Man < 18 years old	18 (2.6)
Have previously used the CrMS within past 6 months for family planning	20 (2.8)
Infertility or past medical procedure to help conceive	335 (47.6)
Vasectomy or tubal ligation	16 (2.3)
Do not expect to be sexually active within 6 months from study entry	88 (12.5)
Planning to conceive at study entry	418 (59.4)
Woman currently pregnant	5 (0.7)
Woman currently taking hormonal birth control	4 (0.6)
Woman had medroxyprogesterone acetate injection within past 2 years and no intervening pregnancy	2 (0.3)
Fewer than two menses in the past 3 months or no menses since stopping hormonal birth control or no menses since last pregnancy	71 (10.1)
Total breastfeeding^a^	37 (5.3)

*^a^Ineligible couples may have multiple reasons for ineligibility*.

*^b^Breastfeeding women were eligible if they were using supplemental feeding and met all other criteria*.

In August 2010, after the study had been enrolling for 1 year and about 100 couples had been enrolled, eligibility criteria were expanded to include couples who were returning to CrM use after 6 months or more without using CrM (for example, after a pregnancy). This change was made to maximize recruitment, while still maintaining a focus on couples who were beginning use of the CrM to avoid pregnancy.

An informed consent document was completed by participants, either on paper or online, and was signed by both partners. If completed on paper, it was signed by the FCP instructing the couple; if completed online, a study staff member called each partner to verify their consent and answer any questions. If the woman did not complete the consent form within 6 weeks of the first CrM follow-up visit or within 6 weeks of the first menstrual flow for postpartum women, they were not enrolled in the study.

### Standard Creighton Model Procedures and Instruments

Couples were instructed in the CrM according to standard procedures. Briefly, couples attended an introductory session (usually in a small group setting), where the scientific underpinnings of the CrM were presented, reading materials were given, and instructions were given for the woman to begin making and interpreting daily vulvar observations. Follow-up sessions were always conducted individually with the couple and a CrM FCP and were recommended to occur about every 2 weeks for the first four follow-ups; subsequent follow-ups were scheduled at longer intervals, for up to eight follow-ups over 1 year. Standard CrM records included the following (22).

- the general intake form, filled out by the couple at entry and containing demographic and reproductive history;
- the CrM user chart (daily diary record) kept by the couple to document vulvar observations, the designation of a day as “fertile” or “infertile” (by use of interpretive stamps), as well as acts of sexual intercourse;
- the CrM follow-up form, a standardized teaching and assessment record used by the CrM FCP during the follow-up sessions;
- an assessment of dimensions of sexual intimacy filled out during two different follow-ups by the couple; and
- CrM pregnancy evaluation forms, described further below under *Outcome*.

Copies of each of these records were obtained and entered into an electronic study database.

### Procedures and Instruments Unique to This Study

- For all new or returning couples for CrM instruction, CrM FCPs filled out a study screening questionnaire, which was used to determine eligibility, as described above (see also Table 1).
- An informed consent document was completed as described above.
- After consent, an entrance questionnaire was completed online, with a separate questionnaire for the woman and the man. This questionnaire contained additional information about demographics and medical history, and questions about childbearing motivations, desires, and behaviors. These include a series of questions which are averaged to form two scaled measures for positive childbearing motivation and negative childbearing motivation. There are also a series of questions about childbearing desires and childbearing intentions. These scales and associated questions have been previously used and validated in other studies (31–34).
- At or near the beginning of each menstrual cycle, a brief cycle questionnaire was completed online, with a separate questionnaire for the woman and the man. This questionnaire assessed intention regarding pregnancy for the coming cycle, and use of any other family planning or fertility tracking methods in the prior cycle, including condoms, withdrawal, urine LH kits, or basal body temperature.
- Upon exit from the study, the couple completed an online exit questionnaire. There were four versions of this questionnaire: for pregnancy or no pregnancy during the study, each separately for the woman and the man. This questionnaire included items about factors around the use of CrM during the study, including repeating questions about use of other family planning or fertility tracking methods. For couples with a pregnancy, it had questions about the pregnancy.

### Outcome

The primary outcome for this study was a clinical pregnancy, as identified by the woman or man. There was active surveillance for pregnancy throughout the study follow-up in three ways: (1) the CrM user chart documented the onset of each menstrual flow and the length of each postovulatory phase (i.e., luteal phase) throughout the study and through the first menstrual flow beyond 1 year whenever possible; (2) the beginning of cycle questionnaire documented the date of the onset of each menstrual flow, independently of the CrM user chart; and (3) the couples attended periodic follow-up sessions with the FCPs. For each identified pregnancy, we reminded and encouraged study participants to contact the CrM FCPs to complete a standard CrM pregnancy evaluation, documented using the CrM pregnancy evaluation form (35). Where circumstances did not allow the original CrM FCP to complete the pregnancy evaluation, we arranged for the study participant to receive a pregnancy evaluation with a different CrM FCP. The CrM pregnancy evaluation form is designed to be completed for every CrM pregnancy. It records dates and results of pregnancy tests, physician encounters, pregnancy symptoms, the couple’s opinion of which day of intercourse resulted in pregnancy and other circumstances of the pregnancy. There is a “long form” and a “short form” version of the pregnancy evaluation form; for the CEIBA study, we requested that the “long form” version should be used. It is a standard CrM procedure for some pregnancies to receive a second pregnancy evaluation by a different CrM FCP (35); in these cases, we collected both pregnancy evaluation forms.

Independently of the pregnancy evaluation form, when pregnancies occurred we sent an online pregnancy version of the study exit questionnaire to both the woman and the man, who completed it directly online, as described above.

### Follow-up and Exit

Couples began to contribute time into the study during the cycle in which they first recorded any sexual intercourse. Some engaged couples were not sexually active at the time they attended the introductory session; they were entered into the active follow-up of the study when they became sexually active, if occurring within 6 months. Couples were followed until 1 year from the first sexual intercourse, voluntary withdrawal from the study, withdrawal by investigators because the couple did not provide data for the study, or pregnancy. Couples were not eligible to enter the study again after pregnancy, regardless of the outcome of the pregnancy.

### Statistical Analysis

We used descriptive statistics to summarize characteristics and reproductive history of study participants at the entrance to the study (and start of CrM use), as well as measures of their childbearing motivations, desires, and intentions; similar methods were used to examine reasons for exclusion from the study for those not eligible. Using the chi-square statistic, we compared demographic and reproductive characteristics of both women and men who were consented versus not consented for the study. We constructed multivariate linear regression models to examine predictors of number of future children desired and number of future children intended, for both women and men. We initially included all variables measured to assess to pregnancy and childbearing motivations; in the final models we only included those variables that were significant to at least *P* = 0.10 in at least one of the final models.

## Results

A total of 1,132 new or returning couples were seen across all centers: 1,090 (96%) were screened, and of those screened, 429 (39%) were eligible couples of which 305 women (71%) consented to the study, along with 290 of their male partners (95%). The number of couples enrolled per month ranged from 0 to 30, with a median of 10 enrollments per month. The 305 women were enrolled across all 17 sites. The minimum number enrolled per sites was 2, the maximum was 60, and the median enrollment per site was 16. Table 1 lists reasons for the 704 women excluded from the study (43 who did not complete screening and 661 who were not eligible), including the reasons for not completing screening or for ineligibility.

Most participants were engaged (39%) or married (51%), college graduates (77% of women), Caucasian non-Hispanic (80% of women), and Catholic (80% of women). There were demographic differences between couples who enrolled in the CEIBA study, and couples seen during the same time who were not enrolled. Women who enrolled were more likely to be of age 20–29 years, to be engaged (versus married or single), to have a college-level education, to have lower household income, to be Catholic, and to have no prior pregnancy or live birth (Table 2). Similar patterns were found for men’s age, marital status, education, and religion. There was a high level of religious involvement: 79% of women and 67% of men reported attending religious or worship service at least once per week. The mean age of women enrolled was 27.8 years.

**Table 2:** Demographic and reproductive characteristics of women and men consented and not consented for the CEIBA study.

	Women			Men		
	Consented *N* (%)	Not consented^a^ *N* (%)	*P*-Value^b^	Consented *N* (%)	Not consented^a^ *N* (%)	*P*-Value^b^
Total *N*	305	827	NA	290	842	NA
**Age at study entrance**						
18–19	2 (0.7)	25 (3.0)	<0.0001	1 (0.3)	2 (0.2)	<0.0001
20–24	93 (30.5)	131 (15.8)		61 (21.0)	76 (9.0)	
25–29	129 (42.3)	225 (27.2)		114 (39.3)	183 (21.7)	
30–34	51 (16.7)	211 (25.5)		68 (23.5)	213 (25.3)	
35–39	14 (4.6)	117 (14.1)		21 (7.2)	134 (15.9)	
40 or more	3 (1.0)	97 (11.7)		15 (5.2)	140 (16.6)	
Missing	13 (4.3)	21 (2.5)		10 (3.5)	95 (11.3)	
**Race and ethnicity**						
Caucasian	245 (80.3)	671 (81.1)	0.8924	247 (85.2)	637 (75.6)	0.001
Hispanic	22 (7.2)	68 (8.2)		13 (4.5)	52 (6.2)	
Others	25 (8.2)	68 (8.2)		19 (6.6)	62 (7.4)	
Missing	13 (4.3)	20 (2.4)		11 (3.8)	92 (10.9)	
**Marital status**						
Single	19 (6.2)	99 (12.0)	<0.0001	18 (6.2)	40 (4.7)	<0.0001
Engaged	118 (38.7)	146 (17.7)		114 (39.3)	148 (17.6)	
Married	155 (50.8)	555 (67.1)		148 (51.0)	563 (66.8)	
Missing	13 (4.3)	27 (3.3)		10 (3.5)	92 (10.9)	
**Years of education**						
HS or less	4 (1.3)	43 (5.2)	0.0248	13 (4.5)	49 (5.8)	0.003
Some college or Voc/Tech	50 (16.4)	146 (17.6)		60 (20.7)	184 (21.8)	
BS/BA	150 (49.2)	370 (44.7)		127 (43.8)	314 (37.3)	
Graduate/professional	86 (28.2)	242 (29.2)		77 (26.6)	194 (23.0)	
Missing	15 (4.9)	26 (3.1)		13 (4.5)	101 (12.1)	
**Annual household income (US dollars)**						
$0–$40,000	86 (28.2)	174 (21.0)	0.0074	NA	NA	NA
$40,001–$60,000	56 (18.4)	132 (16.0)				
$60,001–$80,000	64 (21.0)	168 (20.3)				
Over $80,000	67 (22.0)	256 (31.0)				
Missing	32 (10.5)	97 (11.7)				
**Religion**						
Roman Catholic	244 (80.0)	579 (70.1)	0.0009	206 (71.0)	482 (57.2)	<0.0001
Other Christians^c^	27 (8.9)	123 (14.9)		45 (15.5)	137 (16.3)	
Others^d^	13 (4.3)	77 (9.3)		20 (6.9)	81 (9.6)	
None^e^	7 (2.3)	22 (2.7)		7 (2.4)	43 (5.1)	
Missing	14 (4.6)	26 (3.1)		12 (4.1)	99 (11.8)	
**About how often do you usually attend religious or worship services?^f^**						
Once a week or more	221 (78.7)	NA	NA	177 (66.5)	NA	NA
Less than once a week	34 (12.1)			43 (16.2)		
Never	15 (5.3)			17 (6.4)		
Missing	11 (3.9)			29 (10.9)		
**How important is religion in your life?^f^**						
Important	254 (90.4)	NA	NA	205 (77.1)	NA	NA
Neutral	14 (5.0)			24 (9.0)		
Not important	3 (1.0)			9 (3.4)		
Missing	10 (3.6)			28 (10.5)		
**Number of prior pregnancies**						
0	209 (68.5)	464 (56.1)	0.0007	NA	NA	NA
1	29 (9.5)	124 (15.0)				
2	21 (6.9)	79 (9.6)				
3+	33 (10.8)	134 (16.2)				
Missing	13 (4.3)	26 (3.1)				
**Number of prior live births**						
0	220 (72.1)	546 (66.0)	0.0524	NA	NA	NA
1	23 (7.5)	105 (12.7)				
2	24 (7.9)	68 (8.2)				
3+	25 (8.2)	87 (10.5)				
Missing	13 (4.3)	21 (2.5)				
**Number of prior induced abortions**						
0	286 (93.8)	782 (94.6)	0.4641	NA	NA	NA
1 or more	6 (2.0)	23 (2.8)				
Missing	13 (4.2)	22 (2.7)				
History of any sexually transmitted infection	18 (5.9)	70 (8.5)	0.1531	NA	NA	NA

*NA = not available or not applicable*.

*^a^Not consented includes eligible but not consented couples (*n* = 124), ineligible couples (*n* = 661), and couples for whom eligibility was not determined (*n* = 43)*.

*^b^P-Value is for chi-square comparison of distribution between women consented versus not consented, or men consented versus not consented, respectively. The missing response category is included for the chi-square calculation*.

*^c^Other Christian religion includes Protestant denominations, Christian non-denominational, and Latter-day Saint*.

*^d^Other religion includes Jewish, Buddhism, Islamic, and others*.

*^e^None includes atheist, no religion and agnostic*.

*^f^These two questions are from entrance questionnaire (available only for participants)*.

The most common previously used family planning methods were the oral contraceptive pill (60%) and the male condom (57%) (Table 3). Twenty-two women had previously used the CrM to avoid pregnancy, and 11 women had previously used the CrM to conceive. Overall, there were 27 of all consented women who had previously used the CrM in either way, representing 8.9% of all women enrolled. These women were eligible and enrolled under the expanded criteria (described above) because they were returning to use of the CrM after a pregnancy or other time period of not using the CrM.

**Table 3:** Prior use of family planning methods, reported on the entrance questionnaire (*N* = 273 women).

	Use to avoid *N* (%)	Use to conceive *N* (%)
**Methods of contraception**		
Oral contraceptive	163 (59.7)	NA
Other hormonal methods	41 (15.0)	NA
Male condom	156 (57.1)	NA
Withdrawal	76 (27.8)	NA
Emergency contraception	28 (10.3)	NA
IUD	13 (4.8)	NA
None	73 (26.7)	NA
**Natural family planning or fertility awareness-based methods**		
Creighton model^a^	20 (7.3)	17 (6.2)
Other fertility awareness-based method^b^	102 (37.4)	39 (14.3)
Devices testing for ovulation^c^	15 (5.5)	9 (3.3)
Calendar-based method^d^	30 (11.0)	11 (4.0)
Used breast feeding for delaying of fertility	24 (8.8)	NA
Not having sex for long periods of time (abstinence)	90 (33.0)	NA

*NA, not applicable*.

*^a^Prior Creighton Model use obtained from the screening questionnaire. Last use for family planning had to be at least 6 months prior to screening to be eligible for study*.

*^b^Other fertility awareness based method includes billings ovulation method, symptothermal method (through various organizations or platforms including online), Marquette model, monitoring basal body temperature, and monitoring vaginal secretions*.

*^c^Devices testing for ovulation includes urine ovulation predictor kits, ClearPlan or ClearBlue Fertility Monitor, Persona, Bioself, Ladycomp, Sofia, and Ovulon*.

*^d^Calendar-based method includes Standard Days or CycleBeads, rhythm, other calendar-based method*.

Reasons reported for starting (or resuming) the use of the CrM are indicated in Table 4. For women, the most common reasons were to use a natural method (91%), for moral/ethical/religious reasons (70%), the lack of side effects (71%), or insight into the menstrual cycle and fertility (62%). For men, the most common reasons were to use a natural method (76%), moral/ethical/religious reasons (70%), for wife/partner’s choice (62%), and lack of side effects (61%).

**Table 4:** Women’s and men’s reasons for choosing to learn the Creighton Model Fertility*Care*^TM^ System.

	Female *N* (%)	Male *N* (%)	*P*-Value^a^
Total *N*	272	243	
To use a natural method	248 (91.2)	185 (76.1)	0.006
To use a different natural method	34 (12.5)	13 (5.4)	<0.0001
My partner wants to use this method	69 (25.3)	150 (61.7)	0.0003
To be involved with my partner in family planning	118 (43.2)	144 (59.3)	0.323
Curiosity about natural family planning methods and fertility	80 (29.3)	61 (25.1)	0.020
Friends suggested it	58 (21.3)	32 (13.2)	0.883
Family suggested it	27 (9.9)	23 (9.5)	0.361
Med professional suggested it	27 (10.3)	19 (7.8)	0.591
Religious leader suggested it	109 (39.9)	103 (42.4)	0.033
For moral/ethical/religious reasons	192 (70.3)	149 (61.3)	0.016
Use a method without side effects	192 (70.7)	147 (60.5)	0.095
Avoid med problems with other methods	144 (53.1)	111 (45.7)	0.164
Effectiveness to avoid pregnancy	102 (37.4)	76 (31.3)	0.751
Effectiveness to achieve pregnancy	62 (22.7)	52 (21.4)	0.703
Insight into my (partner’s) menstrual cycle and fertility	168 (61.9)	82 (33.7)	<0.0001
Monitor the menstrual cycle for health	154 (56.8)	69 (28.4)	<0.0001

*^a^Chi-square analysis for proportion of women versus proportion of men*.

Desires, intentions, and motivations for future childbearing are presented in Table 5. At the median, women intended to have three additional children (range 0–11); while men intended to have two additional children (range −2 to 10). About one in five women (21%) intended to have a child within a year, and 60% between 1 and 3 years. The majority of women reported that their closest family members (55%) and closest friends (57%) would be in favor of them having a child at the present time. The mean positive childbearing motivation score for both women and men was 3.3 (range 1–4, with 4 being most positive).

**Table 5:** Women and men’s pregnancy and childbearing motivations and intentions at entry to the CEIBA study.

	Woman, *N* = 272	Woman report man	Man, *N* = 243	Man report woman
How many total children do you want to have? (median, range)	4 (0–11)	3 (0–21)	4 (0–5)	4 (0–10)
How many total children do you actually intend to have? (median, range)	3 (0–9)	3 (0–12)	3 (0–8)	3 (0–8)
[How many children do you want to have in the future]^a^? (median, range)	3 (0–11)	2 (0–21)	3 (0–5)	3 (0–10)
[How many children do you intend to have in the future]^a^? (median, range)	2 (0–9)	2 (0–12)	2 (0–8)	2 (0–8)
How long do you expect to avoid pregnancy? (*N*, %)				
Less than 12 months	33 (12.1)	NA	35 (14.4)	NA
12 months or more	145 (53.3)		126 (51.9)	
Unsure/undecided	19 (7.0)		25 (10.3)	
I want to avoid pregnancy for the rest of my reproductive life	NA		NA	
Missing	75 (27.5)		57 (23.5)	
Desire for a(nother) child at any time in future (*N*, %)				
I definitely want to have a(nother) child	208 (76.4)	204 (75.0)	179 (73.7)	184 (75.7)
I mostly want to have a(nother) child	27 (9.9)	31 (11.4)	20 (8.2)	21 (8.6)
I am not sure whether or not I want to have a(nother) child	20 (7.4)	15 (5.5)	21 (8.6)	15 (6.2)
I mostly do not want to have a(nother) child	10 (3.7)	11 (4.0)	11 (4.5)	12 (4.9)
I definitely do not want to have a(nother) child	5 (1.8)	9 (3.3)	7 (2.9)	6 (2.5)
Missing	2 (0.7)	2 (0.7)	5 (2.1)	5 (2.1)
Intent for a(nother) child at any time in the future (*N*, %)				
I fully intend to have a(nother) child	206 (75.7)	201 (73.9)	177 (72.8)	184 (75.7)
I mostly intend to have a(nother) child	23 (8.5)	28 (10.3)	23 (9.5)	19 (7.8)
I am not sure whether or not I intend to have a(nother) child	20 (7.4)	17 (6.3)	18 (7.4)	18 (7.4)
I mostly intend not to have a(nother) child	14 (5.1)	16 (5.9)	11 (4.5)	10 (4.1)
I fully intend not to have a(nother) child	7 (2.6)	9 (3.3)	9 (3.7)	7 (2.9)
Missing	2 (0.7)	1 (0.4)	5 (2.1)	5 (2.1)
How soon do you want to have a(nother) child? (*N*, %)				
<1 year	62 (22.8)	48 (17.6)	25 (10.3)	45 (18.5)
1–3 year(s)	167 (61.4)	155 (57.0)	157 (64.6)	140 (57.6)
>3 years	30 (11.0)	41 (15.1)	33 (13.6)	31 (12.8)
Do not want to have a(nother) child	20 (7.4)	23 (8.5)	NA	NA
Missing	6 (2.2)	6 (2.2)	28 (11.5)	27 (11.1)
How soon you actually intend to have a(nother) child? (*N*, %)				
<1 year	35 (12.9)	33 (12.1)	26 (10.7)	36 (14.8)
1–3 year(s)	170 (62.5)	168 (61.8)	148 (60.9)	143 (58.8)
>3 years	37 (13.6)	38 (14.0)	30 (12.3)	29 (11.9)
Do not intend to have a(nother) child	22 (8.1)	26 (9.6)	26 (10.7)	NA
Missing	9 (3.3)	8 (2.9)	13 (5.3)	35 (14.4)
How would your closest family members feel about your having a(nother) child at the present time? (*N*, %)				
In favor	154 (56.6)	NA	158 (65.0)	NA
Neutral	50 (18.4)		30 (12.3)	
Against	45 (16.5)		40 (16.5)	
Missing^b^	24 (8.8)		15 (6.2)	
How would your closest family members feel about your having a(nother) child in the future? (*N*, %)				
In favor	243 (89.3)	NA	211 (86.8)	NA
Neutral	17 (6.3)		14 (5.8)	
Against	7 (2.6)		9 (3.7)	
Missing^b^	6 (2.2)		9 (3.7)	
How would your closest friends feel about your having a(nother) child at the present time? (*N*, %)				
In favor	160 (58.8)	NA	149 (61.3)	NA
Neutral	55 (20.2)		53 (21.8)	
Against	29 (10.7)		27 (11.1)	
Missing^b^	29 (10.7)		14 (5.8)	
How would your closest friends feel about your having a(nother) child in the future? (*N*, %)				
In favor	241 (88.6)	NA	204 (84.0)	NA
Neutral	20 (7.4)		25 (10.3)	
Against	4 (1.5)		5 (2.1)	
Missing^b^	8 (2.9)		9 (3.7)	
Childbearing positive motivations score (mean, SD)^c^	3.3 (0.5)	NA	3.3 (0.5)	NA
Childbearing negative motivations score (mean, SD)^c^	2.1 (0.6)	NA	2.4 (0.6)	NA

*^a^This question was not asked directly, but we computed the difference between the number of total children individuals want or intend (from the entrance questionnaire) and the number of children they already have so far*.

^b^The missing category includes “not applicable,” “don’t know,” and “I prefer not to answer this question.”

*^c^The childbearing positive and negative motivation scores are calculated as the mean of 28 and 21 items, respectively. Each combined score is each on a scale from 1 to 4, with 4 denoting the strongest level of motivation*.

We examined factors related to desire for children, with results presented in Table 6. In the each of the models for Tables 6 and 7, we included all variables that were significant in any one of the models. For both women and men, the number of future children desired was positively correlated with positive childbearing motivation scores and perception of spouse or partner’s desired number of future children. Number of future children desired was negatively correlated with age, negative childbearing motivations, number of prior live births, and past use of any hormonal contraception, IUD, or emergency contraception. Marital status was not correlated. Altogether, these variables accounted for over half of the variance (adjusted *R*^2^) for number of future children wanted for both women and men.

**Table 6:** Factors associated with number of children wanted in the future in women and men.

	Women, *N* = 255		Men, *N* = 197	
	β (SE)	*P*-Value	β (SE)	*P*-Value
Age	−0.37 (0.11)	0.001	−0.25 (0.12)	0.041
<25 years				
25–29 years				
>30 years				
Positive childbearing motivations score	0.41 (0.13)	0.002	0.15 (0.19)	0.438
Negative childbearing motivations score	−0.34 (0.13)	0.008	−0.31 (0.16)	0.053
Number of prior live births	−1.26 (0.20)	<0.0001	−0.62 (0.25)	0.014
Married	0.02 (0.17)	0.894	−0.13 (0.20)	0.523
Yes				
No				
How many future children you perceive your partner wants	0.31 (0.04)	<0.0001	0.57 (0.06)	<0.0001
Past use of any hormonal contraception, IUD, or emergency contraception	−0.55 (0.16)	0.001	−0.34 (0.18)	0.060
Adjusted *R*^2^	0.5817		0.5437	
AIC	51.21		45.71	

**Table 7:** Factors associated with number of children intended in the future in women and men.

	Women, *N* = 226		Men, *N* = 194	
	β (SE)	*P*-Value	β (SE)	*P*-Value
Age	−0.15 (0.07)	0.0003	−0.12 (0.08)	0.156
<25 years				
25–29 years				
>30 years				
Positive childbearing motivations score	−0.09 (0.13)	0.333	0.29 (0.13)	0.023
Negative childbearing motivations score	−0.05 (0.09)	0.564	−0.07 (0.11)	0.499
Number of prior live births	−0.20 (0.14)	0.147	−0.48 (0.17)	0.006
Married (versus engaged or other)	−0.28 (0.11)	0.009	−0.40 (0.13)	0.020
Past use of any hormonal contraception, IUD, or emergency contraception	−0.13 (0.10)	0.209	−0.19 (0.12)	0.130
How many future children you want	0.59 (0.04)	<0.0001	0.38 (0.05)	<0.0001
How many future children you perceive your partner wants	0.08 (0.03)	0.002	0.26 (0.05)	<0.0001
Adjusted *R*^2^	0.7579		0.7421	
AIC	−182.23		−114.77	

We also examined factors related to intention for children, with results presented in Table 7. As expected, the number of future children intended was most strongly correlated with number of future children wanted. Positive correlations were also present with perception of number of future children wanted by the spouse or partner, especially for men. There was a negative association for being married (in comparison with mostly engaged couples), and number of prior live births (for men only). For men, there was a positive association with positive childbearing motivations, but no other association with childbearing motivations score for women and men. Altogether, these variables accounted for about 75% of the variance (adjusted *R*^2^) for number of future children intended for both women and men.

## Discussion

We recruited 305 couples to a prospective study of the CrM for family planning. The majority was engaged or married, college graduates, and Catholic. All CrM centers serve women and couples of any faith (or no faith), but the association with Catholic institutions means that the exposure and accessibility to the CrM is greater for Catholic couples. In addition, the majority of women (70%) and men (61%) cited moral/ethical/religious reasons as a motivator for learning the CrM, and many women (40%) and men (42%) indicated that they were motivated by the method being suggested by a religious leader. This is consistent with a study in Germany and Poland which found that, while interest in NFP was not associated with religious motivation, the actual use of NFP was associated with Catholic affiliation (17).

Consistent with eligibility criteria for the study, all couples were originally planning to avoid (or space) pregnancy, but the vast majority had high levels of childbearing motivation, desire, and childbearing intention for the near future. Among women, 69% wanted a child within 3 years, while 71% of men wanted a child within 3 years. It is instructive to compare the positive and negative childbearing motivation scores of the CEIBA couples with those of 401 couples in California in a 1995 study (33). In the California study, all the couples were married: half had no children and had been married for a mean of 3.1 years, and half had one child and had been married for a mean of 4.7 years; also they were somewhat older and wealthier than the couples in the CEIBA study. The mean positive childbearing motivation score in the California study was 2.9 and 2.8 for women and men, respectively. The mean negative childbearing motivation score was 2.2 and 2.4, respectively. The mean total number of children desired was 2.5 for both women and men. In comparison, our sample had higher positive childbearing motivation scores (mean 3.3 for both women and men), lower negative childbearing motivation scores (mean 2.1 and 2.4 for women and men, respectively), and a much higher total number of children wanted (median of 4 for women and 3 for men). This high level of childbearing motivations, desires, and intentions may represent a strong proceptive influence from high levels of involvement in Catholicism and perhaps other religions (36, 37). In addition, CEIBA participants reported that friends and family members were highly supportive of childbearing.

A minority (39%) of screened new users at the participating centers was eligible for the study. Among those who were ineligible, 59% were learning the CrM to try to conceive and 48% had a medical history of infertility. This represents a significant shift from a study conducted more than 10 years ago across eight CrM centers in the United States, which enrolled new CrM users from 1996 to 2000 (38). In that study, only 19% of new users were trying to conceive at study entry. This shift may also be consistent with current trends for the use of fertility apps for smart phones and other personal mobile computing devices, most of which seem to emphasize seeking pregnancy rather than avoiding pregnancy (1).

Because NFP methods can be used either to avoid pregnancy or to conceive, assessing users’ intentions is essential to assessing any NFP method’s effectiveness (12, 13, 18, 39). The CrM educational materials and interactions emphasize radical autonomy for the couple, who decides whether to use it to avoid pregnancy or to conceive (28, 40). Consistent with this philosophy, past evaluations of pregnancy rates with the CrM have been based on behavioral evaluation, rather than stated intentions (25, 28, 29). This contrasts with contraceptive methods that have only the purpose of avoiding pregnancy, and in which prospectively stated intentions have been standard for assessing pregnancy rates (41). One of the purposes of the CEIBA study is to compare intention-based measures of pregnancy rates with behaviorally based measures.

According to the well-established Theory of Reasoned Action and similar theories, intentions are largely driven by desires, and are also influenced by social norms. In turn, desires are largely formed by motivations, as well as other factors (42, 43). Our findings for number of pregnancies wanted and intended by women and men are consistent with this framework. In future analyses, we will assess quantitatively the relationships between desires, intentions, behaviors, and fertility outcomes (i.e., pregnancy) in this study population.

## Conclusion

Women/couples beginning use of the CrM to avoid pregnancy within the United States and Toronto, Canada are mostly young, religious, engaged or newly married, Roman Catholic, and have high levels of motivation, desire, and intention for future childbearing, with the large majority of both women and men wanting a child within 3 years. The CEIBA study assessed these factors at baseline and implemented prospective measures of each of desires, intentions, and sexual/fertility behaviors for up to 1 year after beginning sexual activity. These data will allow us to assess the impact of desires, intentions, and behaviors on the incidence of pregnancy among these couples.

## Ethics Statement

The study was reviewed and approved by the University of Utah Institutional Review Board, IRB number 00034487. All local participating sites either had local IRB approval or an authorization agreement relying upon the University of Utah IRB.

## Author Contributions

JS was the principal investigator for designing and implementing the CEIBA study, directed the data analysis, and wrote the manuscript. CP served as coinvestigator to conduct the study, provided guidance for the analysis, and helped revise the manuscript.

## Conflict of Interest Statement

The authors declare that the research was conducted in the absence of any commercial or financial relationships that could be construed as a potential conflict of interest.
